# Supplementary figures and images for: Construction of a fur null mutant and RNA-sequencing provide deeper global understanding of the Aliivibrio salmonicida Fur regulon
Source: PeerJ. 2017 Jul 13;5:e3461. doi: 10.7717/peerj.3461 (PMC5511505; doi:10.7717/peerj.3461)

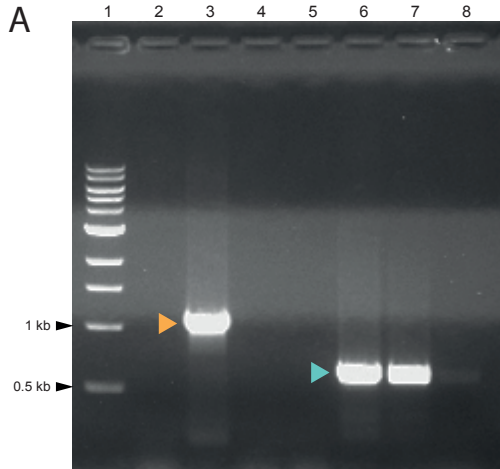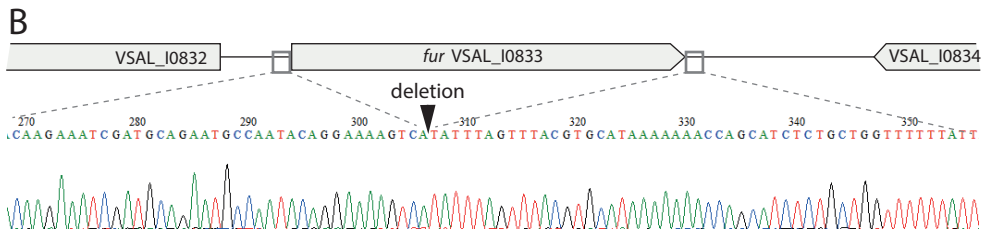

Supplement: Figure S1 — (A) The oligonucleotides FurE and FurF (see Materials and Methods) that anneal to the regions flanking the fur gene were used in PCR amplification to verify the fur deletion. Boiled bacterial samples from agar plates were used as template. Lane 1: 1 kb DNA ladder (from New England Biolabs); Lane 2: negative control (H2O); Lanes 3–5: wild-type colony used in this study with undiluted, 100 × diluted, and 1,000 × diluted template, respectively; Lanes 6–8: same fur null mutant colony used in this study with undiluted, 100 ×, and 1,000 × diluted template, respectively. Orange arrow indicate expected size of PCR product (1,090 bp) from wild-type template that contains the fur gene (444 nt) and flanking sequences. Turquoise arrow indicate expected size of PCR product (640 bp) from fur null mutant template. (B) DNA sequencing chromatogram from Sanger sequencing of the 640 bp PCR product. fur deletion site is indicated by arrowhead and flanking intergenic regions are indicated by dotted lines. [file peerj-05-3461-s001.pdf]

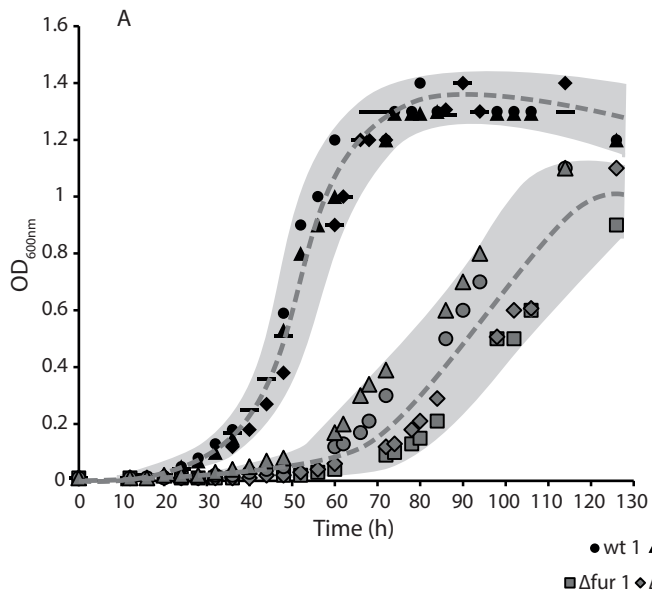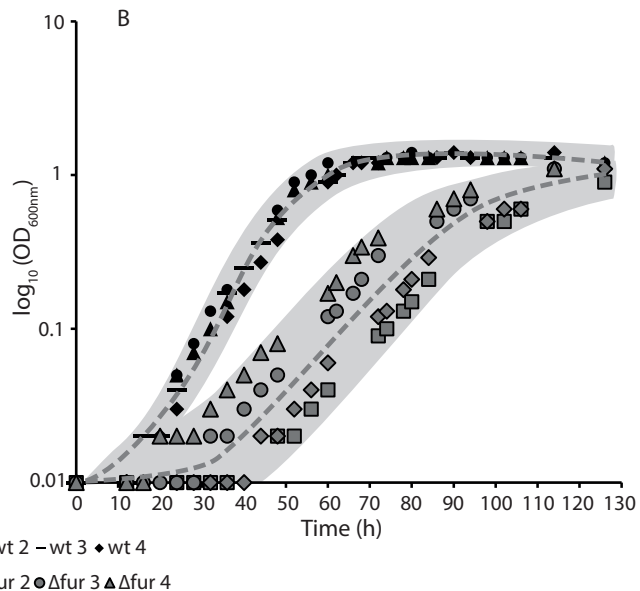

Supplement: Figure S2 — Growth conditions were LB containing 1% NaCl, at 8 °C with 200 rpm agitation. Four biological replicates were used. Grey area indicates the measured variation, and dotted line indicates the average curve. [file peerj-05-3461-s002.pdf]

$\text{H}_2\text{O}_2$ 

2,2'-dipyridyl

wild type

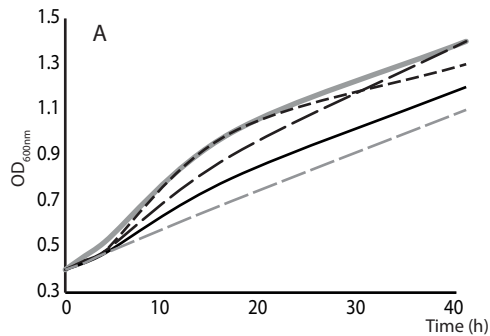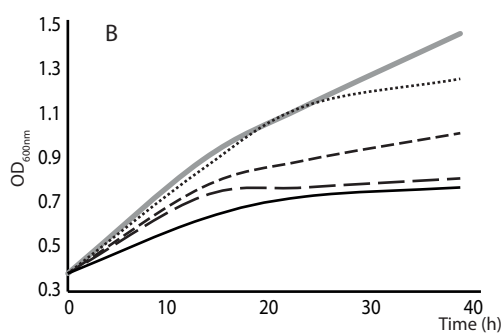 $\Delta fur$ 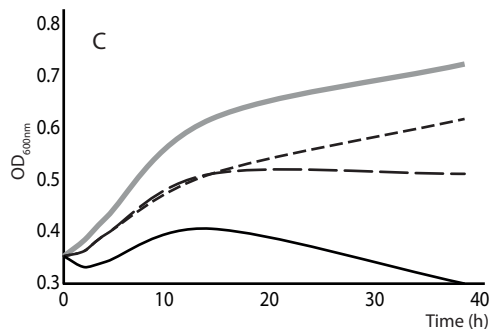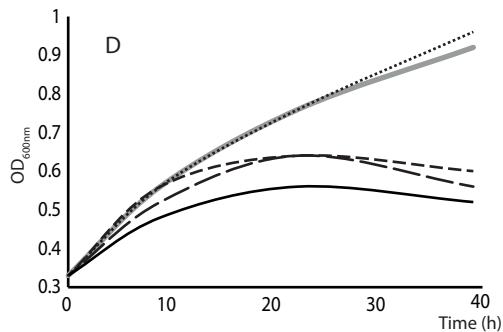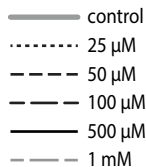

Supplement: Figure S3 — Growth conditions were LB containing 1% NaCl, at 8 °C with 200 rpm agitation. The cultures were grown to mid-log phase, split to smaller cultures and added increasing amounts of H2O2 or 2, 2′-dipyridyl. (A) wt grown with increasing concentrations of H2O2. (B) Δfur grown with increasing concentrations of H2O2. (C) wt grown with increasing concentrations of 2, 2′-dipyridyl. (D) Δfur grown with increasing concentrations of 2, 2′-dipyridyl. [file peerj-05-3461-s003.pdf]
